# Silver(I) diaqua­magnesium *catena*-borodiphosphate(V) monohydrate, AgMg(H_2_O)_2_[BP_2_O_8_]·H_2_O

**DOI:** 10.1107/S1600536811019477

**Published:** 2011-05-28

**Authors:** Hafid Zouihri, Mohammed Saadi, Boujemaa Jaber, Lehcen El Ammari

**Affiliations:** a Centre National pour la Recherche Scientifique et Technique, Division UATRS Angle Allal AlFassi et Avenue des FAR, Hay Ryad, BP 8027, Rabat, Morocco; b Laboratoire de Chimie du Solide Appliquée, Faculté des Sciences, Université Mohammed V-Agdal, Avenue Ibn Batouta, BP 1014, Rabat, Morocco

## Abstract

The title compound contains infinite one-dimensional [BP_2_O_8_]^3−^ borophosphate helical ribbons, built up from alternate BO_4_ and PO_4_ tetra­hedra arranged around the 6_5_ screw axes. The vertex-sharing BO_4_ and PO_4_ tetra­hedra form a spiral ribbon of four-membered rings in which BO_4_ and PO_4_ groups alternate. The ribbons are connected through slightly distorted MgO_4_(H_2_O)_2_ octa­hedra, in which the four O atoms belong to the phosphate groups. The free threads of the helices are occupied by silver ions, which are in an irregular environment surrounded by six O atoms. The central channels of the helices, running along the *c* axis, are filled with the water mol­ecules. The structure is stabilized by O—H⋯O hydrogen bonds between the water mol­ecules and O atoms that are part of the helices. The crystal structure of the title compound is isotopic with other analogous borophosphates such as *A* ^I^ *M* ^II^(H_2_O)_2_[BP_2_O_8_]·H_2_O, where *A* ^I^ = Li, Na, K or NH_4_^+^ and *M* ^II^ = Mg, Mn, Fe, Co, Ni, Cu, Zn or Cd.

## Related literature

For isotypic Mg analogues, see: Kniep *et al.* (1997 ▶); Lin *et al.* (2008 ▶). For other similar borophosphates, see: Kniep *et al.* (1998 ▶); Ewald *et al.* (2007 ▶); Menezes *et al.* (2008 ▶).

## Experimental

### 

#### Crystal data

AgMg(H_2_O)_2_[BP_2_O_8_]·H_2_O
*M* *_r_* = 386.98
Hexagonal, 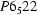

*a* = 9.4577 (4) Å
*c* = 15.8301 (13) Å
*V* = 1226.27 (7) Å^3^
*Z* = 6
Mo *K*α radiation
μ = 2.99 mm^−1^
*T* = 296 K
0.17 × 0.10 × 0.10 mm

#### Data collection

Bruker APEXII CCD detector diffractometer
Absorption correction: multi-scan (*SADABS*; Sheldrick, 1999 ▶) *T*_min_ = 0.705, *T*_max_ = 0.741
18120 measured reflections
1553 independent reflections
1517 reflections with *I* > 2σ(*I*)
*R*_int_ = 0.031

#### Refinement

*R*[*F* ^2^ > 2σ(*F* ^2^)] = 0.037
*wR*(*F* ^2^) = 0.101
*S* = 1.10
1553 reflections
76 parameters
H-atom parameters constrained
Δρ_max_ = 1.59 e Å^−3^
Δρ_min_ = −1.56 e Å^−3^
Absolute structure: Flack (1983 ▶), 543 Friedel pairs
Flack parameter: −0.01 (5)

### 

Data collection: *APEX2* (Bruker, 2005 ▶); cell refinement: *SAINT* (Bruker, 2005 ▶); data reduction: *SAINT*; program(s) used to solve structure: *SHELXS97* (Sheldrick, 2008 ▶); program(s) used to refine structure: *SHELXL97* (Sheldrick, 2008 ▶); molecular graphics: *ORTEP-3 for Windows* (Farrugia, 1997 ▶) and *DIAMOND* (Brandenburg, 2006 ▶); software used to prepare material for publication: *WinGX* (Farrugia, 1999 ▶).

## Supplementary Material

Crystal structure: contains datablocks I, global. DOI: 10.1107/S1600536811019477/fj2421sup1.cif

Structure factors: contains datablocks I. DOI: 10.1107/S1600536811019477/fj2421Isup2.hkl

Additional supplementary materials:  crystallographic information; 3D view; checkCIF report

## Figures and Tables

**Table 1:** Hydrogen-bond geometry (Å, °)

*D*—H⋯*A*	*D*—H	H⋯*A*	*D*⋯*A*	*D*—H⋯*A*
O6—H6*A*⋯O3^i^	0.86	1.89	2.744 (3)	175
O5—H5*A*⋯O5^ii^	0.86	2.06	2.889 (3)	162
O6—H6*B*⋯O2	0.86	1.93	2.781 (3)	170

Symmetry codes: (i) 
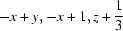
; (ii) 
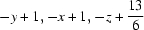
.

**Fig. 1.**
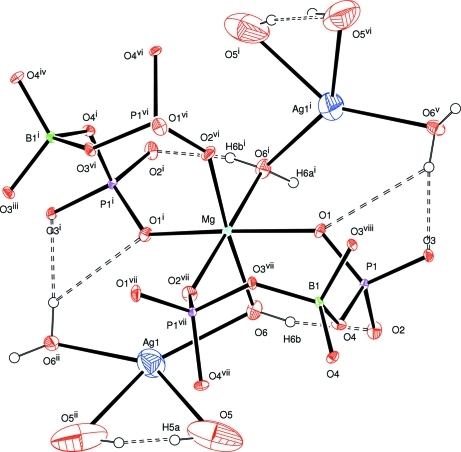
Partial plot of AgMg(H2O)2[BP2O8] H2O crystal structure showing plyhedra linkage. Displacement ellipsoids are drawn at the 50% probability level. Symmetry codes: (i) -y + 1, -x + 1, -z + 13/6; (ii) y - 1, -x + y, z + 1/6; (iii) y - 1, x, -z + 5/3; (iv) x, x-y + 1, -z + 11/6; (v) -x + y - 1, y, -z + 3/2; (vi) -x, -x + y, -z + 4/3; (vii) y, x + 1, -z + 5/3; (viii) x-y + 1, -y + 2, -z + 2.

**Fig. 2.**
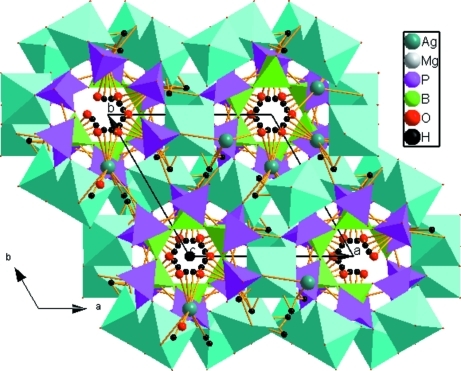
Projection view of the AgMg(H2O)2[BP2O8] H2O framework structure showing tunnel running along c direction where water molecules are located.

## supplementary crystallographic information

### Comment

The rich structural chemistry of the borophosphates system has generated considerable contemporary interest as a consequence of the interesting physical and chemical properties of the porous or tunnel structures generally adopted by the inorganic solids which are formed (Kniep *et al.*, 1998, Ewald *et al.*, 2007). Most of these compounds were synthesized with alkali (A^I^) and transition metal cations (*M*^II^), with the general formula A^I^*M*^II^(H_2_O)_2_[BP_2_O_8_].H_2_O, under hydrothermal conditions at 443–463 K (Kniep *et al.* (1997) and Lin *et al.* (2008)).

The crystal structure of the new synthesized helical borophosphate-hydrate AgMg(H_2_O)_2_[BP_2_O_8_].H_2_O is isotopic with other analogues borophosphates like A^I^*M*^II^ (H_2_O)_2_[BP_2_O_8_].H_2_O (A^I^ = Li, Na, K, NH_4_^+^ and *M*^II^ = Mg, Mn, Fe, Co, Ni, Cu, Zn, Cd) (Menezes *et al.*, (2008)). Fig. 1 represents the plot of the asymmetric unit showing the cationic environment and the connection between different polyhedra. The BO_4_ and PO_4_ tetrahedra are relatively regular with B—O and P—O bond lengths ranging from 1.455 (3) Å to 1.480 (3) Å and from 1.503 (2) Å to to 1.569 (2) Å, respectively. Whereas, in the distorted MgO_4_(H_2_O)_2_ octahedron, the distances Mg—O vary between 2.053 (2) Å and 2.169 (3) Å. Moreover, the AgO_6_ polyhedron is more irregular with Ag—O distances in the range of 2.462–2.725 (3) Å.

The structure consists of infinite one dimensional helical anionic ribbons [BP_2_O_8_]^3-^ constructed by corner-sharing BO_4_ and PO_4_ tetrahedra, arranged around the 6_5_ screw axes. The ribbons borders are connected with Mg^2+^ cations *via* the terminal oxygen atoms of the phosphate groups. A three dimensional network is formed by interconnection between the (AgO_6_)_n_ helices running along [001] and the tetrahedral ribbons *via* the slightly distorted MgO_4_(H_2_O)_2_ octahedra. The central channels of the helices, running along *c* axis, are filled up with the water molecule as shown in Fig 2. The structure is stabilized by O—H···O hydrogen bonds between water molecules and O atoms that are part of the helices (Table 1).

### Experimental

The compound was hydrothermally synthesized at 453 °K for 7 days in a 25 ml Teflon-lined steel autoclave from the mixture of MgO, H_3_BO_3_, H_3_PO_4_ (85%), AgNO_3_ and 5 ml of distilled water in the molar ratio of 1:4:6:1:165. The brilliant colourless octahedral crystals were recovered and washed with hot water, then dried in air. Except for boron and hydrogen the presence of the elements were additionally confirmed by EDAX measurements.

### Refinement

The highest peak in the difference map is at 0.76 Å from Ag1 atom, and the minimum peak is at 0.52 Å from Ag1 atom.

### Crystal data

**Table:**

AgMg(H_2_O)_2_[BP_2_O_8_]·H_2_O	*D*_x_ = 3.144 Mg m^−^^3^
*M**_r_* = 386.98	Mo *K*α radiation, λ = 0.71073 Å
Hexagonal, *P*6_5_22	Cell parameters from 1553 reflections
Hall symbol: P 65 2 ( 0 0 1)	θ = 2.5–33.0°
*a* = 9.4577 (4) Å	µ = 2.99 mm^−^^1^
*c* = 15.8301 (13) Å	*T* = 296 K
*V* = 1226.27 (7) Å^3^	Prism, colourless
*Z* = 6	0.17 × 0.10 × 0.10 mm
*F*(000) = 1128	

### Data collection

**Table:**

Bruker APEXII CCD detector diffractometer	1553 independent reflections
Radiation source: fine-focus sealed tube	1517 reflections with *I* > 2σ(*I*)
graphite	*R*_int_ = 0.031
ω and φ scans	θ_max_ = 33.0°, θ_min_ = 2.5°
Absorption correction: multi-scan (*SADABS*; Sheldrick, 1999)	*h* = −14→14
*T*_min_ = 0.705, *T*_max_ = 0.741	*k* = −14→13
18120 measured reflections	*l* = −24→24

### Refinement

**Table:**

Refinement on *F*^2^	Hydrogen site location: inferred from neighbouring sites
Least-squares matrix: full	H-atom parameters constrained
*R*[*F*^2^ > 2σ(*F*^2^)] = 0.037	*w* = 1/[σ^2^(*F*_o_^2^) + (0.0472*P*)^2^ + 4.7811*P*] where *P* = (*F*_o_^2^ + 2*F*_c_^2^)/3
*wR*(*F*^2^) = 0.101	(Δ/σ)_max_ < 0.001
*S* = 1.10	Δρ_max_ = 1.59 e Å^−^^3^
1553 reflections	Δρ_min_ = −1.56 e Å^−^^3^
76 parameters	Extinction correction: *SHELXL97* (Sheldrick, 2008), Fc^*^=kFc[1+0.001xFc^2^λ^3^/sin(2θ)]^-1/4^
0 restraints	Extinction coefficient: 0.0049 (7)
Primary atom site location: structure-invariant direct methods	Absolute structure: Flack (1983), 543 Friedel pairs
Secondary atom site location: difference Fourier map	Flack parameter: −0.01 (5)

### Special details

**Table:**

Geometry. All e.s.d.'s (except the e.s.d. in the dihedral angle between two l.s. planes) are estimated using the full covariance matrix. The cell e.s.d.'s are taken into account individually in the estimation of e.s.d.'s in distances, angles and torsion angles; correlations between e.s.d.'s in cell parameters are only used when they are defined by crystal symmetry. An approximate (isotropic) treatment of cell e.s.d.'s is used for estimating e.s.d.'s involving l.s. planes.
Refinement. Refinement of *F*^2^ against ALL reflections. The weighted *R*-factor *wR* and goodness of fit *S* are based on *F*^2^, conventional *R*-factors *R* are based on *F*, with *F* set to zero for negative *F*^2^. The threshold expression of *F*^2^ > 2σ(*F*^2^) is used only for calculating *R*-factors(gt) *etc*. and is not relevant to the choice of reflections for refinement. *R*-factors based on *F*^2^ are statistically about twice as large as those based on *F*, and *R*- factors based on ALL data will be even larger.

### Fractional atomic coordinates and isotropic or equivalent isotropic displacement parameters (Å^2^)

**Table:**

	*x*	*y*	*z*	*U*_iso_*/*U*_eq_	
Ag1	0.18579 (4)	0.81421 (4)	1.0833	0.03836 (17)	
Mg	0.10244 (16)	0.55122 (8)	0.9167	0.0065 (2)	
P1	0.16930 (8)	0.78125 (8)	0.75206 (4)	0.00471 (13)	
B1	−0.1513 (2)	0.6974 (5)	0.7500	0.0052 (6)	
O1	0.1362 (3)	0.6230 (3)	0.79151 (13)	0.0098 (4)	
O2	0.3176 (3)	0.9319 (3)	0.78429 (13)	0.0103 (4)	
O3	0.1801 (3)	0.7639 (3)	0.65405 (12)	0.0070 (3)	
O4	0.0211 (2)	0.8082 (2)	0.76691 (12)	0.0067 (3)	
O5	0.1244 (8)	1.0000	1.0000	0.082 (3)	
H5A	0.0727	0.9715	1.0473	0.098*	
O6	0.2931 (3)	0.7990 (3)	0.94403 (14)	0.0129 (4)	
H6A	0.3869	0.8124	0.9579	0.016*	
H6B	0.3096	0.8516	0.8974	0.016*	

### Atomic displacement parameters (Å^2^)

**Table:**

	*U*^11^	*U*^22^	*U*^33^	*U*^12^	*U*^13^	*U*^23^
Ag1	0.0451 (3)	0.0451 (3)	0.0305 (3)	0.0267 (3)	−0.00288 (19)	−0.00288 (19)
Mg	0.0067 (5)	0.0063 (4)	0.0066 (5)	0.0034 (3)	0.000	0.0011 (4)
P1	0.0044 (2)	0.0053 (2)	0.0044 (2)	0.0023 (2)	0.0003 (2)	0.0011 (2)
B1	0.0057 (11)	0.0073 (14)	0.0030 (13)	0.0036 (7)	0.0004 (10)	0.000
O1	0.0145 (10)	0.0083 (9)	0.0072 (8)	0.0062 (7)	0.0010 (7)	0.0031 (7)
O2	0.0054 (8)	0.0108 (9)	0.0102 (8)	0.0008 (7)	−0.0015 (6)	−0.0002 (7)
O3	0.0103 (8)	0.0089 (8)	0.0038 (7)	0.0062 (7)	0.0009 (6)	0.0011 (6)
O4	0.0039 (7)	0.0065 (8)	0.0095 (8)	0.0023 (6)	−0.0015 (6)	−0.0025 (6)
O5	0.042 (2)	0.072 (5)	0.140 (8)	0.036 (3)	−0.033 (3)	−0.066 (6)
O6	0.0099 (9)	0.0136 (10)	0.0130 (9)	0.0041 (8)	−0.0003 (7)	0.0031 (7)

### Geometric parameters (Å, °)

**Table:**

Ag1—O6^i^	2.461 (2)	P1—O2	1.503 (2)
Ag1—O6	2.462 (2)	P1—O4	1.563 (2)
Ag1—O5	2.487 (4)	P1—O3	1.569 (2)
Ag1—O5^i^	2.487 (4)	B1—O4	1.455 (3)
Ag1—Mg	3.4363 (4)	B1—O4^v^	1.455 (3)
Ag1—Mg^i^	3.4363 (4)	B1—O3^vi^	1.480 (3)
Mg—O2^ii^	2.053 (2)	B1—O3^ii^	1.480 (3)
Mg—O2^iii^	2.053 (2)	O2—Mg^vii^	2.053 (2)
Mg—O1	2.067 (2)	O3—B1^vi^	1.480 (3)
Mg—O1^iv^	2.067 (2)	O5—Ag1^viii^	2.487 (4)
Mg—O6	2.169 (3)	O5—H5A	0.8600
Mg—O6^iv^	2.169 (3)	O6—H6A	0.8600
P1—O1	1.503 (2)	O6—H6B	0.8600
			
O6^i^—Ag1—O6	131.89 (11)	O2^iii^—Mg—O6^iv^	89.01 (10)
O6^i^—Ag1—O5	148.09 (10)	O1—Mg—O6^iv^	83.08 (9)
O6—Ag1—O5	79.32 (11)	O1^iv^—Mg—O6^iv^	85.88 (9)
O6^i^—Ag1—O5^i^	79.32 (11)	O6—Mg—O6^iv^	87.91 (14)
O6—Ag1—O5^i^	148.09 (10)	O1—P1—O2	115.72 (13)
O5—Ag1—O5^i^	71.0 (2)	O1—P1—O4	110.01 (12)
O2^ii^—Mg—O2^iii^	94.25 (15)	O2—P1—O4	106.40 (12)
O2^ii^—Mg—O1	99.94 (9)	O1—P1—O3	107.40 (12)
O2^iii^—Mg—O1	90.53 (9)	O2—P1—O3	110.86 (12)
O2^ii^—Mg—O1^iv^	90.53 (9)	O4—P1—O3	106.05 (11)
O2^iii^—Mg—O1^iv^	99.94 (9)	O4—B1—O4^v^	102.9 (3)
O1—Mg—O1^iv^	164.64 (15)	O4—B1—O3^vi^	113.29 (11)
O2^ii^—Mg—O6	89.01 (10)	O4^v^—B1—O3^vi^	112.88 (11)
O2^iii^—Mg—O6	175.52 (10)	O4—B1—O3^ii^	112.88 (11)
O1—Mg—O6	85.88 (9)	O4^v^—B1—O3^ii^	113.29 (11)
O1^iv^—Mg—O6	83.08 (9)	O3^vi^—B1—O3^ii^	102.0 (3)
O2^ii^—Mg—O6^iv^	175.52 (10)	H6A—O6—H6B	104.9

Symmetry codes: (i) −*y*+1, −*x*+1, −*z*+13/6; (ii) *y*−1, −*x*+*y*, *z*+1/6; (iii) *y*−1, *x*, −*z*+5/3; (iv) *x*, *x*−*y*+1, −*z*+11/6; (v) −*x*+*y*−1, *y*, −*z*+3/2; (vi) −*x*, −*x*+*y*, −*z*+4/3; (vii) *y*, *x*+1, −*z*+5/3; (viii) *x*−*y*+1, −*y*+2, −*z*+2.

### Hydrogen-bond geometry (Å, °)

**Table:**

*D*—H···*A*	*D*—H	H···*A*	*D*···*A*	*D*—H···*A*
O6—H6A···O3^ix^	0.86	1.89	2.744 (3)	175
O5—H5A···O5^i^	0.86	2.06	2.889 (3)	162
O6—H6B···O2	0.86	1.93	2.781 (3)	170

Symmetry codes: (ix) −*x*+*y*, −*x*+1, *z*+1/3; (i) −*y*+1, −*x*+1, −*z*+13/6.
